# Impact of Serum Chemerin Levels on Liver Functional Reserves and Platelet Counts in Patients with Hepatocellular Carcinoma

**DOI:** 10.3390/ijms150711294

**Published:** 2014-06-25

**Authors:** Kenji Imai, Koji Takai, Tatsunori Hanai, Makoto Shiraki, Yusuke Suzuki, Hideki Hayashi, Takafumi Naiki, Youichi Nishigaki, Eiichi Tomita, Masahito Shimizu, Hisataka Moriwaki

**Affiliations:** 1 Department of Gastroenterology/Internal Medicine, Gifu University Graduate School of Medicine, 1-1 Yanagido, Gifu 501-1194, Japan; E-Mails: koz@gifu-u.ac.jp (K.T.); hanai0606@yahoo.co.jp (T.H.); mshiraki-gif@umin.ac.jp (Mak.S.); shimim-gif@umin.ac.jp (Mas.S.); hmori@gifu-u.ac.jp (H.M.); 2 Department of Gastroenterology, Gifu Municipal Hospital, 7-1 Kashima-cho, Gifu 500-8323, Japan; E-Mails: usukesuzuki0915@yahoo.co.jp (Y.S.); hide-hayashi@umin.ac.jp (H.H.); naiki-gif@umin.ac.jp (T.N.); nishigak@he.mirai.ne.jp (Y.N.); etomita_jp@yahoo.co.jp (E.T.)

**Keywords:** Chemerin, hepatocellular carcinoma, liver functional reserve, prognosis, recurrence

## Abstract

Obesity-related metabolic abnormalities, including adipokine imbalance and chronic inflammation, are involved in liver carcinogenesis. Chemerin, a novel adipokine, plays a critical role in adipogenesis, energy metabolism, and inflammation. We evaluated the impact of serum chemerin levels on liver functional reserves in hepatocellular carcinoma (HCC) patients and on the recurrence and prognosis of HCC. This study included 44 patients with any stage of HCC who underwent curative treatment at Gifu Municipal Hospital (Gifu, Japan) between 2006 and 2007. Recurrence-free survival and overall survival were estimated using the Kaplan-Meier method. Serum albumin levels (Pearson’s correlation coefficient; *r* = 0.3110, *p* = 0.0399), platelet counts (*r* = 0.4159, *p* = 0.0050), and prothrombin times (*r* = 0.3775, *p* = 0.0115) were significantly correlated with serum chemerin levels in patients with HCC, and they were inversely correlated with Child-Pugh scores (*r* = −0.3732, *p* = 0.0126), serum alanine aminotransferase levels (*r* = −0.3864, *p* = 0.0105), and total bilirubin levels (*r* = −0.4023, *p* = 0.0068). Among these variables, a multiple comparison test identified that platelet counts and total bilirubin levels were associated with serum chemerin levels (*p* < 0.0083). No significant correlation was found between serum chemerin levels and recurrence-free survival (*p* = 0.3691) or overall survival (*p* = 0.7916). In HCC patients, serum chemerin concentrations were correlated with liver functional reserves and platelet counts, but not with recurrence or prognosis.

## 1. Introduction

Hepatocellular carcinoma (HCC) is one of the most frequently occurring cancers worldwide. In addition to conventional risk factors, such as persistent infection with hepatitis viruses and alcohol consumption, recent epidemiological and clinical studies have revealed that obesity and its related metabolic disorders, including diabetes mellitus, are also major risk factors for HCC development [1,2,3]. Obesity-induced pathophysiological conditions present insulin resistance, chronic inflammation, excess oxidative stress, and adipokine imbalance, and are considered to link obesity and liver carcinogenesis [3,4,5]. For instance, insulin resistance and higher levels of serum leptin, a pro-inflammatory adipokine that is elevated in obese individuals, are significantly associated with an increased risk of HCC recurrence after curative treatment [4,6].

Chemerin is an adipocyte-secreted protein with autocrine/paracrine roles in adipose tissue development and function [7,8]. Chemerin and its major receptor, CMKLR1, are expressed in white adipose tissue, and circulating levels of this adipokine are elevated in obese humans and rodents [9,10]. Furthermore, the levels of chemerin in serum and adipose tissue are significantly increased in patients with metabolic syndrome (Mets) compared to healthy controls [11,12]. Patients with nonalcoholic fatty liver disease (NAFLD), which is the major hepatic manifestation of obesity and Mets, also demonstrate increased levels of circulating chemerin [13,14]. Conversely, exercise-induced weight loss decreases serum chemerin levels and is associated with improvements in Mets biomarkers, such as insulin resistance, fasting glucose levels, and visceral fat volume [15]. These reports indicate that chemerin is an adipokine that links obesity and metabolic disorders.

In addition to regulating endocrine function in obesity and metabolism, chemerin plays a critical role in the inflammatory process [16]. High levels of tissue chemerin and CMKLR1-expressing cells have been found in various human inflammatory diseases, such as rheumatoid arthritis and inflammatory ascites [17,18]. Increased serum chemerin levels are positively correlated with levels of circulating pro-inflammatory cytokines, tumor necrosis factor-α, and interleukin-6, as well as leptin [13,19]. Moreover, a recent clinical study showed that serum chemerin concentrations were significantly elevated in patients with chronic hepatitis C [20], but its levels in patients with HCC have not yet been clarified.

In this study, we measured serum chemerin concentrations in HCC patients and examined whether they were correlated with liver functional reserves. In addition, we designed a prospective case-series analysis to examine recurrence-free survival and overall survival in patients with HCC of any stage who had received curative treatment, and stratified the outcomes according to baseline serum chemerin concentrations.

## 2. Results and Discussion

### 2.1. Patient Baseline Characteristics and Laboratory Data

The baseline characteristics and laboratory data for the 44 patients (29 men and 15 women; median age, 71 years) are shown in Table 1. The median serum chemerin level for all patients with HCC was 130.5 ng/mL (range, 80–312 ng/mL). The numbers of patients with Child-Pugh scores of 5, 6, 7, and 8 were 9, 22, 7, and 6, respectively. The Child-Pugh classification is a scoring system used to determine the prognosis of cirrhosis, based on five clinical parameters [21], including the presence of ascites (1, none; 2, mild; 3, moderate-severe), encephalopathy (1, none; 2, grade I–II; 3, grade III–IV), serum albumin level (1, >3.5 g/dL; 2, 2.8–3.5 g/dL; 3, <2.8 g/dL), total bilirubin levels (1, <2 mg/dL; 2, 2.0–3.0 mg/dL; 3, >3 mg/dL), and prothrombin time (1, >70%; 2, 40%–70%; 3, <40%). A total score of 5–6, 7–9, and 10–15 is defined as Child-Pugh class A, B, and C, respectively. In the present study, 31 patients were classified into Child-Pugh class A, 13 patients into class B, and none into class C. The median serum chemerin levels for Child-Pugh class A and class B in these patients were 135.0 ng/mL (range, 80–312 ng/mL) and 128.0 ng/mL (range, 87.1–160 ng/mL), respectively, and there was no significant difference between these values (*p* = 0.1146); however, those with Child-Pugh scores of 5, 6, 7, and 8 were 154 ng/mL (range, 92.6–312 ng/mL), 133 ng/mL (range, 80–220 ng/mL), 129 ng/mL (range, 102–160 ng/mL), and 103 ng/mL (range, 87.1–132 ng/mL), respectively, showing an overall significant difference (*p* = 0.0126).

**Table 1:** Baseline demographic and clinical characteristics.

Variables	*n* = 44
Sex (male/female)	29/15
Age (years)	71 (50–82)
Etiology (B/C/B + C/other)	4/38/1/1
BMI (kg/m^2^)	22.5 (15.6–33.5)
Child-Pugh score (5/6/7/8)	9/22/7/6
ALB (g/dL)	3.5 (2.6–4.5)
ALT (IU/L)	46 (12–146)
T-Bil (mg/dL)	1.0 (0.5–3.7)
PLT (×10^4^/μL)	9.5 (3.6–18.8)
PT (%)	70 (50–100)
FPG (mg/dL)	100 (74–224)
HbA_1c_ (%)	5.0 (3.6–9.4)
AFP (ng/dL)	32.5 (1.7–16931)
PIVKA-II (mAU/mL)	26.5 (5–1860)
Tumor size (cm)	1.9 (1.0–15.3)
Tumor number (1/2/3/4/5)	30/9/2/2/1
Stage (I/II/III)	17/21/6
Initial treatment (resection/RFA/TACE + RFA)	1/38/5
Chemerin (ng/mL)	130.5 (80–312)

Values are presented as medians (range). AFP, alpha-fetoprotein; ALB, albumin; ALT, alanine aminotransferase; BMI, body mass index; FPG, fasting plasma glucose; HbA_1c_, hemoglobin A_1c_; PIVKA-II, proteins induced by vitamin K absence or antagonist-II; PLT, platelet counts; PT, prothrombin time; RFA, radiofrequency ablation; TACE, transarterial chemoembolization; T-Bil, total bilirubin.

### 2.2. Association of the Serum Chemerin Level with Liver Functional Reserve and Other Clinical Indexes

**Figure 1:**
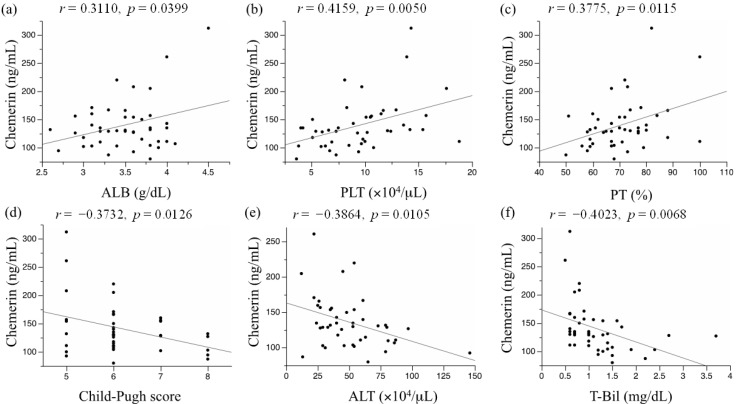
Association between serum chemerin levels and (**a**) serum albumin level; (**b**) platelet count; (**c**) prothrombin time; (**d**) Child-Pugh score; (**e**) serum alanine aminotransferase level; and (**f**) serum total bilirubin level.

We evaluated the correlation of serum chemerin levels with various laboratory data using Pearson regression analysis (Table 2). Among the tested variables, serum albumin levels (Pearson’s Correlation Coefficient; *r* = 0.3110, *p* = 0.0399) (Figure 1a), platelet counts (*r* = 0.4159, *p* = 0.0050) (Figure 1b), and prothrombin times (*r* = 0.3775, *p* = 0.0115) (Figure 1c) were significantly correlated with serum chemerin concentrations. On the other hand, the Child-Pugh scores (*r* = −0.3732, *p* = 0.0126) (Figure 1d), serum alanine aminotransferase (ALT) levels (*r* = −0.3864, *p* = 0.0105) (Figure 1e), and total bilirubin levels (*r* = −0.4023, *p* = 0.0068) (Figure 1f) were inversely correlated with serum chemerin levels. Among these variables, a Bonferroni multiple comparison test identified platelet counts and total bilirubin levels as parameters significantly associated with serum chemerin levels (*p* < 0.0083, Table 2). In addition, among chemerin and the six clinical variables as described above (serum albumin levels, platelet counts, prothrombin times, Child-Pugh scores, serum ALT levels, and total bilirubin levels), only the serum chemerin levels remained as a variable significantly correlated with all other six variables (Supplementary Table S1). Of these correlations, Bonferroni test indicated that correlation coefficient between chemerin and platelet count or total bilirubin had *p* value < 0.0071 (overall *p* < 0.1).

Furthermore, multiple linear regression analysis was used to test whether the six variables listed in Figure 1 showed independent correlations with serum chemerin levels. Among these variables, we excluded serum levels of albumin and bilirubin and prothrombin times because they are parameters for the determination of the Child-Pugh scores and thus, might strongly interact with the scores (see Supplementary Table S1). Therefore, four variables, including an objective variable (chemerin level) and three explanatory variables (Child-Pugh score, ALT levels, and platelet counts), were retained in the multivariate model. Stepwise regression and a maximum *R*^2^ approach indicated that Child-Pugh scores and serum ALT levels were independently correlated with serum chemerin levels, and the following regression equation was obtained (Equation (1)). The variables were treated as raw data prior to testing, regardless of their normal distribution; the degrees of freedom were calculated based on the number of objective and explanatory variables, and the stepwise procedure was conducted using JMP^®^ 10 (SAS Institute Inc., Cary, NC, USA).

[Serum chemerin levels] = 262.0 − 15.2 × (Child-Pugh scores) − 0.61 × (serum ALT levels) F-statistic = 8.28 on 2 and 40 degrees of freedom(1)

Serum chemerin levels and tumor-related factors, including tumor numbers, tumor sizes, or serum levels of alpha-fetoprotein (AFP) and proteins induced by vitamin K absence or antagonist-II (PIVKA-II), did not correlate significantly. The median serum chemerin levels for tumor sizes less than 1.5 cm (10 patients) and greater than 2.0 cm (20 patients) were 130.5 ng/mL (range, 100.0–312.0 ng/mL) and 132.0 ng/mL (range, 92.6–261.0 ng/mL), respectively, and no significant difference was found between these values (*p* = 0.4949). Furthermore, the serum chemerin levels were not correlated with the clinical stage of HCC (*p* = 0.2596); the median serum chemerin levels for Stages I (17 patients), II (21 patients), and III (6 patients) were 130.0 ng/mL (range, 87.1–312.0 ng/mL), 129.0 ng/mL (range, 80.0–205.0 ng/mL), and 151.0 ng/mL (range, 92.6–261.0 ng/mL), respectively.

**Table 2:** Correlation of the serum chemerin level with various laboratory data by Pearson regression analysis.

Parameters	Pearson’s Correlation Coefficient	*p* Value
Age	0.2294	0.1389
BMI	0.2040	0.1895
Child-Pugh score	−0.3732	0.0126 *
ALB (g/dL)	0.3110	0.0399 *
ALT (IU/L)	−0.3864	0.0105 *
T-Bil (mg/dL)	−0.4023	0.0068 *^,†^
PLT (×10^4^/μL)	0.4159	0.0050 *^,†^
PT (%)	0.3775	0.0115 *
FPG (mg/dL)	−0.1145	0.4761
HbA_1c_ (%)	−0.0509	0.7750
FIRI (mg/dL)	−0.2217	0.2226
HOMA-IR	−0.1093	0.4963
AFP (ng/dL)	−0.1764	0.2698
PIVKA-II (mAU/mL)	0.0493	0.7689
Tumor number	−0.1100	0.4773
Tumor size (cm)	−0.0510	0.7423
d-ROM (Carr U)	−0.0427	0.7830
BAP (μmol/L)	−0.3591	0.2782
Leptin (ng/mL)	0.0805	0.6032
Visfatin (ng/mL)	−0.0181	0.9074
Resistin (ng/mL)	0.0832	0.5913
Vaspin (ng/mL)	−0.1180	0.4457

* *p* < 0.05 by single regression analysis; ^†^ *p* < 0.0083 by Bonferroni-corrected comparisons; AFP, alpha-fetoprotein; ALB, albumin; ALT, alanine aminotransferase; BAP, biological anti-oxidant potential; BMI, body mass index; d-ROM, derivatives of reactive oxygen metabolites; FIRI, fasting immunoreactive insulin; FPG, fasting plasma glucose; HbA_1c_, hemoglobin A_1c_; HOMA-IR, homeostasis model assessment of insulin resistance; PIVKA-II, proteins induced by vitamin K absence or antagonist-II; PLT, platelet counts; PT, prothrombin time; T-Bil, total bilirubin.

Neither obesity- or diabetes-related factors, including body mass index (BMI), fasting plasma glucose (FPG) levels, fasting immunoreactive insulin (FIRI) levels, hemoglobin A1c (HbA1c), and homeostasis model assessment of insulin resistance (HOMA-IR), were significantly correlated with the serum chemerin concentrations. In addition, serum derivatives of reactive oxygen metabolites (d-ROM) levels or other adipokines, including leptin, visfatin, resistin, and vaspin, did not show significant associations with serum chemerin levels (Table 2).

### 2.3. Impact of the Serum Chemerin Levels on Recurrence-Free Survival and Overall Survival in Patients with Hepatocellular Carcinoma (HCC)

**Figure 2:**
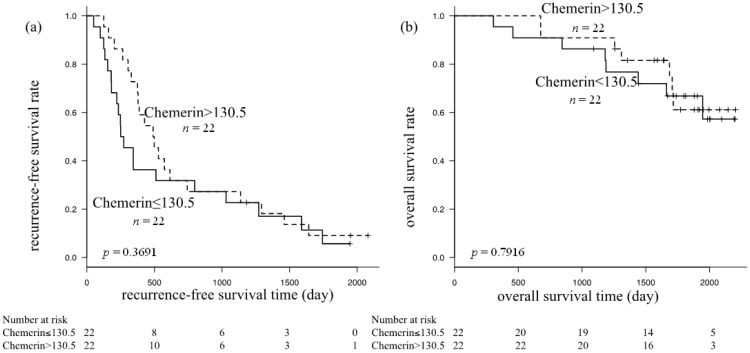
Kaplan-Meier curves for (**a**) recurrence-free survival; and (**b**) overall survival in subgroups based on serum chemerin levels; neither showed a significant difference by the log-rank test.

We next evaluated the impact of the serum chemerin level on recurrence-free survival and overall survival in HCC patients using the Kaplan-Meier method and log-rank test. Statistical analyses were performed after the 44 patients were divided into two subgroups, based on the median serum chemerin concentration (≤130.5 or >130.5 ng/mL). There was no significant difference in recurrence-free survival (*p* = 0.3691) (Figure 2a) or overall survival (*p* = 0.7916) (Figure 2b) between these two groups.

### 2.4. Discussion

We demonstrated for the first time that the serum chemerin levels declined in parallel with the worsening liver functional reserves in HCC patients. This observation may be partly explained by the fact that the liver is one of the main chemerin-secreting organs [22,23,24], although adipose tissue produces more. We further found that higher levels of serum chemerin may directly reflect liver functional reserves rather than BMI and other diabetes-related factors, which were reported to raise circulating chemerin levels [10,25]. A recent study showed that serum chemerin levels were significantly higher in patients with chronic hepatitis C, but, controversially, the levels were negatively associated with the necro-inflammatory grades [20]. On the other hand, serum chemerin was significantly elevated in patients with non-alcoholic steatohepatitis compared with those with NAFLD [26], indicating that measuring serum chemerin levels might be useful for evaluating the hepatic inflammatory grade. In addition to hepatic inflammation, the results of the present study suggest that the liver functional reserves might be assessed by serum chemerin measurements, at least in patients with chronic hepatitis or liver cirrhosis with HCC.

Moreover, the results of this study also showed that serum chemerin levels are significantly correlated with platelet counts in HCC patients. This is another important finding of the present study because thrombocytopenia, which is a frequent feature in patients with liver cirrhosis, serves as an indicator of liver fibrosis and portal hypertension [27,28]. Platelet counts are widely used for evaluating the progression of liver fibrosis, which is a critical predictive factor for liver cirrhosis [27,28]. Whether serum chemerin levels are histologically associated with liver fibrosis grade should be examined in a future study.

The results of this study provide the first evidence that serum chemerin levels are not associated with the recurrence of HCC or overall survival in patients with this malignancy. Significant correlations were not detected between serum chemerin levels and increased oxidative stress, hyperleptinemia, or hypervisfatinemia, which are known to associate with early recurrence and HCC stage progression [5,6,29]. Moreover, there were no significant correlations between serum chemerin levels and insulin resistance-related factors, including FPG, FIRI, HbA_1c_, and HOMA-IR. These observations are important because insulin resistance is a critical risk factor for recurrence of HCC after curative treatment [4]. On the other hand, several studies have shown evidence that chemerin has a great effect on glucose homeostasis, and may play a direct role in increasing insulin sensitivity and glucose uptake [30,31], whereas conflicting results have also been provided [32]. Diabetes mellitus or insulin resistance often exist in liver cirrhosis [33], and serum chemerin is also markedly elevated in chronic hepatitis C patients [20]. Future investigations are required to determine whether chemerin promotes or suppresses the onset of diabetes and insulin resistance in patients with chronic liver disease.

The impact of chemerin on other carcinogenesis remains controversial. Serum chemerin levels were significantly higher in gastric cancer patients than in healthy volunteers, and its elevation has been correlated with advanced clinical stage [34]. Overexpression of chemerin mRNA and protein was also associated with tumor angiogenesis and poor clinical outcomes in patients with squamous cell carcinoma of the tongue [35]. Conversely, a recent clinical trial demonstrated that the expression of the chemerin protein was significantly decreased in HCC tissue compared with noncancerous liver tissue, and that the lower expression of this adipokine was associated with a poor prognosis [36]. Interestingly, the expression level of chemerin was significantly correlated with the infiltration of dendritic cells and natural killer cells into HCC tissue [36], indicating that chemerin might exert suppressive effects on the development and progression of HCC via activation of antitumor immunity. Long-term follow-up studies examining the impact of serum chemerin levels on the development of initial HCC are ongoing.

There are several limitations in the present study. One of the most critical limitations is that we did not compare serum levels of chemerin between HCC patients and healthy persons. We also need to examine the possibility that chemerin might also correlate with albumin, platelet, and total bilirubin levels in healthy persons. Therefore, a comparative study that clarifies these points is currently in progress. The next limitation is that the form of chemerin was not been determined in this study. Chemerin is secreted as an inactive precursor that requires proteolytic cleavage for activation, and relevant proteases that can activate chemerin are enriched in the cancer microenvironment [37,38]. Therefore, knowing the amount of circulating chemerin present in HCC patients in a bioactive form or as an inactive precursor is important. Further studies that quantify the levels of bioactive chemerin or the activity of specific proteases activating chemerin should be pursued.

## 3. Materials and Methods

### 3.1. Patients and Measurement of Serum Chemerin Concentration

A total of 84 primary HCC patients with any stage had undergone initial treatment for HCC at Gifu Municipal Hospital (Gifu, Japan) between 2006 and 2007. Among them, we evaluated 44 patients whose therapies were judged to be curative. Tumor stage was defined according to the system of the Liver Cancer Study Group of Japan [39]. HCC nodules were detected using imaging modalities, including dynamic computed tomography (CT), dynamic magnetic resonance imaging (MRI), and abdominal arteriography. The guidelines [39], described above, suggest that HCC with increased arterial blood flow should be the main target of diagnosis and treatment. This suggestion is based on the fact that intranodular blood supply is correlated with the histological degree of differentiation (e.g., dysplastic nodule to well-differentiated HCC, moderately or poorly differentiated HCC) [40]. Therefore, HCC was diagnosed based on a typical hypervascular tumor stain on angiography and typical dynamic study findings of enhanced staining in the early phase and attenuation in the delayed phase.

Fasting serum samples were collected at the time of diagnosis, and serum chemerin concentrations were assessed, in duplicate, by using an enzyme-linked immunosorbent assay (BioVendor R&D, Brno, Czech Republic). All patients provided informed consent. This study was approved by research ethics boards of our hospital according to good clinical practice, the declaration of Helsinki, and applicable regulations.

### 3.2. Treatment and Follow-Up Strategy

**Figure 3:**
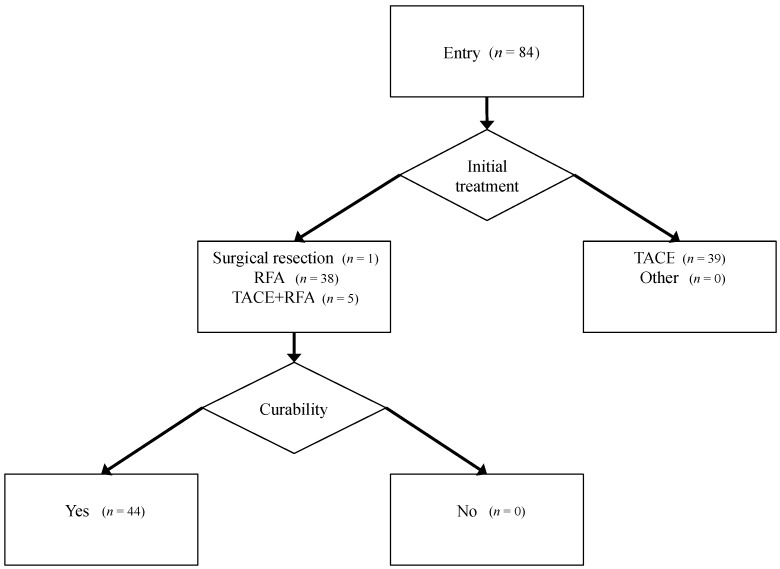
Patient flow, treatment selection, and curability.

One patient was treated with surgical resection, 38 with radiofrequency ablation (RFA), and five with RFA after transarterial chemoembolization, according to the Clinical Practice Guidelines for HCC issued by the Japan Society of Hepatology [39]. In these 44 patients, therapeutic effects were judged to be curative by complete disappearance of the imaging characteristics of HCC on dynamic CT or MRI. The flowchart of patient enrollment, selected treatment, and curability of treatment is shown in Figure 3.

After discharge, patients were followed, on an outpatient basis by monthly assessments of the levels of serum tumor markers, such as alpha-fetoprotein (AFP) or proteins induced by vitamin K absence or antagonist-II (PIVKA-II), and by imaging modalities, such as abdominal ultrasonography, dynamic CT scanning, or dynamic MRI, every three months. Recurrent HCC was diagnosed, using these imaging modalities, as the appearance of another lesion, which showed the typical image of HCC, as described above, in a different segment from the primary one. Recurrence-free survival and overall survival were defined as intervals from the date of initial treatment until the date of recurrence or death, respectively, or until April 2009 for non-recurrence or surviving patients, respectively.

### 3.3. Statistical Analysis

The Pearson product-moment correlation coefficient was used for measuring the linear correlation between two continuous variables. Recurrence-free survival and overall survival were estimated using the Kaplan-Meier method, and differences between curves were evaluated using the log-rank test. Statistical significance was defined as *p* < 0.05.

## 4. Conclusions

Serum chemerin concentrations are positively correlated with serum albumin levels, platelet counts, and prothrombin times, but are inversely correlated with Child-Pugh scores, serum ALT levels, and total bilirubin levels, indicating that serum chemerin may reflect liver functional reserves in chronic hepatitis and liver cirrhosis patients with HCC. Serum chemerin levels have no significant correlation with recurrence and prognosis for HCC patients.

## Supplementary Files

Supplementary File 1

## References

[1] Muto Y., Sato S., Watanabe A., Moriwaki H., Suzuki K., Kato A., Kato M., Nakamura T., Higuchi K., Nishiguchi S. (2006). Overweight and obesity increase the risk for liver cancer in patients with liver cirrhosis and long-term oral supplementation with branched-chain amino acid granules inhibits liver carcinogenesis in heavier patients with liver cirrhosis. Hepatol. Res..

[2] Sun B., Karin M. (2012). Obesity, inflammation, and liver cancer. J. Hepatol..

[3] Shimizu M., Tanaka T., Moriwaki H. (2013). Obesity and hepatocellular carcinoma: Targeting obesity-related inflammation for chemoprevention of liver carcinogenesis. Semin. Immunopathol..

[4] Imai K., Takai K., Nishigaki Y., Shimizu S., Naiki T., Hayashi H., Uematsu T., Sugihara J., Tomita E., Shimizu M. (2010). Insulin resistance raises the risk for recurrence of stage I hepatocellular carcinoma after curative radiofrequency ablation in hepatitis C virus-positive patients: A prospective, case series study. Hepatol. Res..

[5] Suzuki Y., Imai K., Takai K., Hanai T., Hayashi H., Naiki T., Nishigaki Y., Tomita E., Shimizu M., Moriwaki H. (2013). Hepatocellular carcinoma patients with increased oxidative stress levels are prone to recurrence after curative treatment: A prospective case series study using the d-ROM test. J. Cancer Res. Clin. Oncol..

[6] Watanabe N., Takai K., Imai K., Shimizu M., Naiki T., Nagaki M., Moriwaki H. (2011). Increased levels of serum leptin are a risk factor for the recurrence of stage I/II hepatocellular carcinoma after curative treatment. J. Clin. Biochem. Nutr..

[7] Roh S.G., Song S.H., Choi K.C., Katoh K., Wittamer V., Parmentier M., Sasaki S. (2007). Chemerin—A new adipokine that modulates adipogenesis via its own receptor. Biochem. Biophys. Res. Commun..

[8] Rourke J.L., Dranse H.J., Sinal C.J. (2013). Towards an integrative approach to understanding the role of chemerin in human health and disease. Obes. Rev..

[9] Goralski K.B., McCarthy T.C., Hanniman E.A., Zabel B.A., Butcher E.C., Parlee S.D., Muruganandan S., Sinal C.J. (2007). Chemerin, a novel adipokine that regulates adipogenesis and adipocyte metabolism. J. Biol. Chem..

[10] Bozaoglu K., Bolton K., McMillan J., Zimmet P., Jowett J., Collier G., Walder K., Segal D. (2007). Chemerin is a novel adipokine associated with obesity and metabolic syndrome. Endocrinology.

[11] Dong B., Ji W., Zhang Y. (2011). Elevated serum chemerin levels are associated with the presence of coronary artery disease in patients with metabolic syndrome. Intern. Med..

[12] Min J.L., Nicholson G., Halgrimsdottir I., Almstrup K., Petri A., Barrett A., Travers M., Rayner N.W., Mägi R., Pettersson F.H. (2012). Coexpression network analysis in abdominal and gluteal adipose tissue reveals regulatory genetic loci for metabolic syndrome and related phenotypes. PLoS Genet..

[13] Sell H., Divoux A., Poitou C., Basdevant A., Bouillot J.L., Bedossa P., Tordjman J., Eckel J., Clément K. (2010). Chemerin correlates with markers for fatty liver in morbidly obese patients and strongly decreases after weight loss induced by bariatric surgery. J. Clin. Endocrinol. Metab..

[14] Yilmaz Y., Yonal O., Kurt R., Alahdab Y.O., Eren F., Ozdogan O., Celikel C.A., Imeryuz N., Kalayci C., Avsar E. (2011). Serum levels of omentin, chemerin and adipsin in patients with biopsy-proven nonalcoholic fatty liver disease. Scand. J. Gastroenterol..

[15] Saremi A., Shavandi N., Parastesh M., Daneshmand H. (2010). Twelve-week aerobic training decreases chemerin level and improves cardiometabolic risk factors in overweight and obese men. Asian J. Sports Med..

[16] Wittamer V., Franssen J.D., Vulcano M., Mirjolet J.F., Le Poul E., Migeotte I., Brézillon S., Tyldesley R., Blanpain C., Detheux M. (2003). Specific recruitment of antigen-presenting cells by chemerin, a novel processed ligand from human inflammatory fluids. J. Exp. Med..

[17] Parolini S., Santoro A., Marcenaro E., Luini W., Massardi L., Facchetti F., Communi D., Parmentier M., Majorana A., Sironi M. (2007). The role of chemerin in the colocalization of NK and dendritic cell subsets into inflamed tissues. Blood.

[18] Vermi W., Riboldi E., Wittamer V., Gentili F., Luini W., Marrelli S., Vecchi A., Franssen J.D., Communi D., Massardi L. (2005). Role of ChemR23 in directing the migration of myeloid and plasmacytoid dendritic cells to lymphoid organs and inflamed skin. J. Exp. Med..

[19] Gu P., Jiang W., Lu B., Shi Z. (2013). Chemerin is associated with inflammatory markers and metabolic syndrome phenotypes in hypertension patients. Clin. Exp. Hypertens..

[20] Kukla M., Zwirska-Korczala K., Gabriel A., Waluga M., Warakomska I., Szczygiel B., Berdowska A., Mazur W., Wozniak-Grygiel E., Kryczka W. (2010). Chemerin, vaspin and insulin resistance in chronic hepatitis C. J. Viral Hepat..

[21] Pugh R.N., Murray-Lyon I.M., Dawson J.L., Pietroni M.C., Williams R. (1973). Transection of the oesophagus for bleeding oesophageal varices. Br. J. Surg..

[22] Krautbauer S., Wanninger J., Eisinger K., Hader Y., Beck M., Kopp A., Schmid A., Weiss T.S., Dorn C., Buechler C. (2013). Chemerin is highly expressed in hepatocytes and is induced in non-alcoholic steatohepatitis liver. Exp. Mol. Pathol..

[23] Takahashi M., Takahashi Y., Takahashi K., Zolotaryov F.N., Hong K.S., Kitazawa R., Iida K., Okimura Y., Kaji H., Kitazawa S. (2008). Chemerin enhances insulin signaling and potentiates insulin-stimulated glucose uptake in 3T3-L1 adipocytes. FEBS Lett..

[24] Weigert J., Neumeier M., Wanninger J., Filarsky M., Bauer S., Wiest R., Farkas S., Scherer M.N., Schäffler A., Aslanidis C. (2010). Systemic chemerin is related to inflammation rather than obesity in type 2 diabetes. Clin. Endocrinol..

[25] Tonjes A., Fasshauer M., Kratzsch J., Stumvoll M., Bluher M. (2010). Adipokine pattern in subjects with impaired fasting glucose and impaired glucose tolerance in comparison to normal glucose tolerance and diabetes. PLoS One.

[26] Kukla M., Zwirska-Korczala K., Hartleb M., Waluga M., Chwist A., Kajor M., Kajor M.C., Berdowska A., Wozniak-Grygiel E., Buldak R. (2010). Serum chemerin and vaspin in non-alcoholic fatty liver disease. Scand. J. Gastroenterol..

[27] Afdhal N., McHutchison J., Brown R., Jacobson I., Manns M., Poordad F., Weksler B., Esteban R. (2008). Thrombocytopenia associated with chronic liver disease. J. Hepatol..

[28] Chou R., Wasson N. (2013). Blood tests to diagnose fibrosis or cirrhosis in patients with chronic hepatitis C virus infection: A systematic review. Ann. Intern. Med..

[29] Ninomiya S., Shimizu M., Imai K., Takai K., Shiraki M., Hara T., Tsurumi H., Ishizaki S., Moriwaki H. (2011). Possible role of visfatin in hepatoma progression and the effects of branched-chain amino acids on visfatin-induced proliferation in human hepatoma cells. Cancer Prev. Res..

[30] Ernst M.C., Haidl I.D., Zuniga L.A., Dranse H.J., Rourke J.L., Zabel B.A., Butcher E.C., Sinal C.J. (2012). Disruption of the *chemokine-like receptor-1* (*CMKLR1*) gene is associated with reduced adiposity and glucose intolerance. Endocrinology.

[31] Takahashi M., Okimura Y., Iguchi G., Nishizawa H., Yamamoto M., Suda K., Kitazawa R., Fujimoto W., Takahashi K., Zolotaryov F.N. (2011). Chemerin regulates beta-cell function in mice. Sci. Rep..

[32] Kralisch S., Weise S., Sommer G., Lipfert J., Lossner U., Bluher M., Stumvoll M., Fasshauer M. (2009). Interleukin-1β induces the novel adipokine chemerin in adipocytes *in vitro*. Regul. Pept..

[33] Kingston M.E., Ali M.A., Atiyeh M., Donnelly R.J. (1984). Diabetes mellitus in chronic active hepatitis and cirrhosis. Gastroenterology.

[34] Wang C., Wu W.K., Liu X., To K.F., Chen G.G., Yu J., Ng E.K. (2013). Increased serum chemerin level promotes cellular invasiveness in gastric cancer: A clinical and experimental study. Peptides.

[35] Wang N., Wang Q.J., Feng Y.Y., Shang W., Cai M. (2013). Overexpression of chemerin was associated with tumor angiogenesis and poor clinical outcome in squamous cell carcinoma of the oral tongue. Clin. Oral Investig..

[36] Lin W., Chen Y.L., Jiang L., Chen J.K. (2011). Reduced expression of chemerin is associated with a poor prognosis and a lowed infiltration of both dendritic cells and natural killer cells in human hepatocellular carcinoma. Clin. Lab..

[37] Yamaguchi Y., Du X.Y., Zhao L., Morser J., Leung L.L. (2011). Proteolytic cleavage of chemerin protein is necessary for activation to the active form, Chem157S, which functions as a signaling molecule in glioblastoma. J. Biol. Chem..

[38] Zabel B.A., Allen S.J., Kulig P., Allen J.A., Cichy J., Handel T.M., Butcher E.C. (2005). Chemerin activation by serine proteases of the coagulation, fibrinolytic, and inflammatory cascades. J. Biol. Chem..

[39] (2010). Clinical Practice Guidelines for hepatocellular carcinoma—The Japan Society of Hepatology 2009 update. Hepatol. Res..

[40] Hayashi M., Matsui O., Ueda K., Kawamori Y., Kadoya M., Yoshikawa J., Gabata T., Takashima T., Nonomura A., Nakanuma Y. (1999). Correlation between the blood supply and grade of malignancy of hepatocellular nodules associated with liver cirrhosis: Evaluation by CT during intraarterial injection of contrast medium. Am. J. Roentgenol..

