# Supplementary material for: Impact of Serum Chemerin Levels on Liver Functional Reserves and Platelet Counts in Patients with Hepatocellular Carcinoma
Source: Int J Mol Sci. 2014 Jun 25;15(7):11294–306. doi: 10.3390/ijms150711294 (PMC4139783; doi:10.3390/ijms150711294)
Supplement: Supplementary File 1 [file ijms-15-11294-s001.pdf]

Supplementary Information

Table S1. Correlation coefficients among chemerin and other six clinical variables that were correlated with serum chemerin level by Pearson regression analysis.

Variable	Child-Pugh Score	ALB	ALT	T-Bil	PLT	PT	Chemerin
**Child-Pugh score**		−0.5785 *,††	−0.0692	0.5158 *,††	−0.3399 *	−0.6703 *,††	−0.3732 *
**ALB**			−0.0579	−0.0229	0.2238	0.2835	0.3110 *
**ALT**				0.2077	−0.1543	−0.0267	−0.3864 *
**T-Bil**					−0.3690 *	−0.4259 *	−0.4023 *,†
**PLT**						0.3601 *	0.4159 *,†
**PT**							0.3775 *
**Chemerin**							

* $p < 0.05$; †† $p < 0.0036$ (overall $p < 0.05$); † $p < 0.0071$ (overall $p < 0.1$) by Bonferroni-corrected comparisons.
